# Widespread Distribution and Adaptive Degradation of Microcystin Degrader (*mlr*-Genotype) in Lake Taihu, China

**DOI:** 10.3390/toxins13120864

**Published:** 2021-12-03

**Authors:** Chenlin Hu, Yanxia Zuo, Liang Peng, Nanqin Gan, Lirong Song

**Affiliations:** 1 State Key Laboratory of Fresh Water Ecology and Biotechnology, Institute of Hydrobiology, Chinese Academy of Sciences, Wuhan 430072, China; chu7@uh.edu (C.H.); yxzuo@ihb.ac.cn (Y.Z.); tpengliang@jnu.edu.cn (L.P.); 2 Graduate School of Chinese Academy of Sciences, Beijing 100049, China; 3 College of Pharmacy, University of Houston, Houston, TX 77204, USA; 4 Institute of Hydrobiology, Jinan University, Guangzhou 510632, China

**Keywords:** microcystin, biodegradation, *mlrA*, distribution, adaptation, *Microcystis*

## Abstract

Microbial degradation is an important route for removing environmental microcystins (MCs). Here, we investigated the ecological distribution of microcystin degraders (*mlr*-genotype), and the relationship between the substrate specificity of the microcystin degrader and the profile of microcystin congener production in the habitat. We showed that microcystin degraders were widely distributed and closely associated with *Microcystis* abundance in Lake Taihu, China. We characterized an indigenous degrader, *Sphingopyxis* N5 in the northern Lake Taihu, and it metabolized six microcystin congeners in increasing order (RR > LR > YR > LA > LF and LW). Such a substrate-specificity pattern was congruent to the order of the dominance levels of these congeners in northern Lake Taihu. Furthermore, a meta-analysis on global microcystin degraders revealed that the substrate-specificity patterns varied geographically, but generally matched the profiles of microcystin congener production in the degrader habitats, and the indigenous degrader typically metabolized well the dominant MC congeners, but not the rare congeners in the habitat. This highlighted the phenotypic congruence between microcystin production and degradation in natural environments. We theorize that such congruence resulted from the metabolic adaptation of the indigenous degrader to the local microcystin congeners. Under the nutrient microcystin selection, the degraders might have evolved to better exploit the locally dominant congeners. This study provided the novel insight into the ecological distribution and adaptive degradation of microcystin degraders.

## 1. Introduction

Freshwater cyanobacterial *Microcystis* blooms can produce potent liver toxin microcystin that presents a serious threat to global public health. In the natural environment, microcystins can be degraded by certain microbes [1,2,3,4]. The production and degradation of microcystins are a pair of important ecological phenomena. However, compared to substantial studies of microcystin producers, our understanding of the eco-physiology of microcystin degraders remains relatively limited.

Since the first microcystin degrader (*Sphingomonas* sp. ACM-3962) was reported in 1994 [5], tens of microcystin degraders have been documented and have been recently reviewed [1,2,3,4,6], and the most common belongs to the sphingomonad group (such as *Sphingopyxis* and *Sphingomonas*). Although various mechanisms have been implied for microcystin degradation [7,8,9,10], only the *mlr* gene-mediated pathway has been demonstrated to date [11,12,13,14]. This pathway comprises of at least three intracellular hydrolytic enzymes (MlrABC) and a putative transporter (MlrD). The transmembrane microcystinase MlrA first cleaves cyclic microcystin into a linear form within the periplasm [15], causing significant reduction in the toxicity [16,17], and the other enzymes (e.g., linearized-microcystinase MlrB, tetrapeptidease MlrC etc.) subsequently cleave the linearized microcystin into shorter peptides, which can be finally metabolized into CO_2_ [12,13,14,16]. Among various *mlr*-genotype microcystin degraders isolated, the most common still belongs to sphingomonad members. Because the gene *mlrA* is unique to the microcystin degrader [18], the *mlrA*-specific primers and probe have been developed as the molecular tool for detecting and monitoring microcystin degraders [18,19]. In the microcystin-occurring environment, the *mlr*-genotype and non-*mlr*-genotype degraders typically coexist [20], their relative contribution to removing environmental microcystin remains an open question. However, an increasing number of studies have reported isolation of the sphingomonad degraders (*mlr*-genotype) from microcystin-occurring environments in China [12,21,22,23,24,25,26], Japan [27,28,29,30], Thailand [31], Spain [20], Poland [32], Canada [33], Australia [5,34,35], and New Zealand [36]. Studies also demonstrated the ecological relevance of *mlr*-genotype degraders in removing environmental microcystins [34,37], whereas the non-*mlr*-genotype degraders were suggested to be the dominant degraders in some lakes of Europe [9,38] and North America [7,39,40]. Recently, multiple studies have started to explore the temporal dynamics of *mlr*-genotype microcystin degraders in Asian and European water bodies where massive toxic *Microcystis* blooms occur, e.g., in the Japanese Lake Suwa [37,41], the Chinese Lake Dianchi [42], the Spanish Reservoir San Juan [20,43]. However, only a few locations were surveyed, a picture of the large-scale spatial distribution of microcystin degraders (*mlrA*-genotype) has yet to be depicted specifically.

Additionally, there exists a gap regarding to the substrate specificity of the indigenous microcystin degraders for various microcystin congeners (such as MC-LR, MC-RR, and MC-YR). A certain microcystin-utilizing degrader often mineralizes these congeners at differential rates [28,35,44]. So far, few scholars have studied the eco-physiological mechanism by which the microcystin degrader develops such substrate specificity. The substrate specificity is an important biological factor that affects the environmental fate of the substrate congeners, and has been extensively studied in the bioremediation of the organic pollutants e.g., aromatic compounds, polychlorinated biphenyls (PCBs), and herbicides over the past few decades [45]. At an evolutionary level, the substrate specificity has been theorized to result from the long-term natural selection imposed by the availability of local substrate congener in the metabolizer habitat [46], in other words, the metabolizer has evolved to specifically metabolize better these substrates that were initially abundant in the ecosystem; and at the molecular level, it is associated with adaptive genetic mutations in the metabolic enzymes [46,47,48]. As for the indigenous microcystin degrader, is its substrate specificity also associated with the dominance level of native microcystin congener in the degrader habitat? Or is there the metabolic adaptation to the native dominant microcystin congeners? In a water body there indeed exists a relatively stable composition of microcystin congeners, with only certain ones being perennially dominant due to the existence of the stable dominant genotypes of microcystin production [49]. In response to such perennially stable composition of native microcystin congeners, have the indigenous microcystin degraders evolved to exploit these dominant ones at higher rates than these minor ones? Or is there the phenotypic congruence between the substrate-specificity pattern of the degrader for microcystin congeners and the profile pattern of microcystin congener production in the degrader habitat? These questions have yet to be addressed but are important for establishing the eco-physiological and molecular mechanisms of the substrate specificity for microcystin congeners.

In such a context, this work aimed to (i) investigate the distribution of microcystin degraders (*mlr*-genotype) in a large Chinese Lake Taihu where *Microcystis* blooms annually occur; (ii) explore the relationship between the substrate specificity of the indigenous degrader and the profile pattern of native microcystin congeners in the degrader habitat. 

## 2. Results

### 2.1. Physiochemical and Biological Parameters in Lake Taihu

The major nutrient (total nitrogen and phosphorus) and chlorophyll *a* (Chl *a*) in Lake Taihu over 2008 and 2009 have been previously reported and were in the ranges of 1.6–3.6 mg/L, 0.10–0.26 mg/L, and14.1–212.2 μg/L, respectively [50]. This study further summarized the four major parameters (temperature, pH, dissolved O_2_, and *Microcystis* abundance), which were only available in the samples collected from 7 sampling sites (N1, N2, N7, W1, W3, S1, and S6), specifically, temperature (2–22 °C, mean = 9.4 °C), pH (7.4–9.1, mean = 8.1), and dissolved O_2_ (7.5–14.2 mg/L, mean = 10.7 mg/L) as well as *Microcystis* abundance (33–69,552 cell/mL, mean = 15,577 cell/mL).

### 2.2. Large-Scale Distribution of mlrA-Genotype Bacteria in Lake Taihu

**Figure 1:**
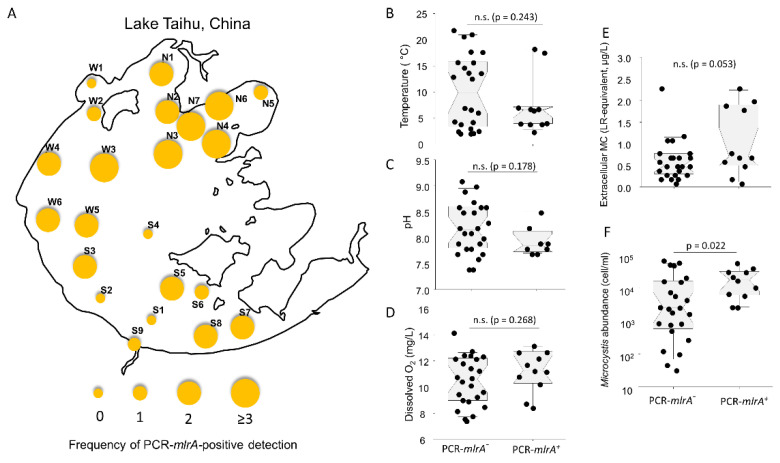
This is a (**A**) Large-scale spatial distribution of *mlr*-genotype (measured by the frequency of positive PCR-*mlrA* detection) in Lake Taihu from November 2008 to March 2009. (**B**–**F**) Notched box plots showing the difference in the five parameters (temperature, pH, dissolved O_2_, extracellular microcystin, and *Microcystis* abundance) between negative PCR-*mlrA* (PCR-*mlr*^-^) and positive PCR-*mlrA* (PCR-*mlrA*^+^) group (Two-sample *t*-test, n.s., not significant, *p* > 0.05).

The present survey showed that *mlrA*-genotype bacteria were widely distributed in Lake Taihu (Figure 1A). The *mlrA*-genotype bacteria were detected in 18 sites out of 22 survey sites (82%). These four sites with complete *mlrA*-negative results are S1, S2, S4, and W1, most of which were located in the most southern lake region. Additionally, the average frequency (2.1) of PCR-*mlrA*-positive detection in the northern lake sites (*n* = 11, N1–7, and W1–4) was higher than that (1.3) in southern lake sites (*n* = 11, W5–6, S1–9) (Figure S1), such spatial pattern in the frequency of positive PCR-*mlrA* detection was consistent with that in the severity of both eutrophication and algal blooms in northern and southern Lake Taihu as documented elsewhere [51,52].

To explore whether the presence of the *mlrA* genotype was statistically significantly correlated with certain physiochemical parameters, we focused on the 35 samples in the 7 sampling sites (N1, N2, N7, W1, W3, S1, and S6) where physiochemical and algal data were available, and we divided these samples into two groups: PCR-*mlrA*-positive and PCR-*mlrA*-negative sample group, respectively (Figure 1B–F). It was found that the average of *Microcystis* abundance (2654–57,751 cell/mL, mean = 20,733 cell/mL) in the PCR-*mlrA*-positive samples were significantly higher than that (33–69,592 cell/mL, mean = 13,214 cell/mL) in the PCR-*mlrA*-negative samples (*p* = 0.022, Figure 1F), the average of extracellular microcystin level (0.1–2.3 µg/L, mean = 1.1 µg/L) in the PCR-*mlrA*-positive samples were also higher than that (0.1–2.3 µg/L, mean = 0.6 µg/L) in the PCR-*mlrA*-negative samples, but statistically insignificant with the *p*-value of 0.053 (Figure 1E). No significant difference in the physicochemical parameters (temperature, pH, and dissolved O_2_) were observed between the PCR-*mlrA*-positive and PCR-*mlrA*-negative group (*p* > 0.15, Figure 1B,C). Taken together, all these data highlighted a close association of *mlrA*-genotype bacteria with *Microcystis* abundance.

### 2.3. Isolation and Identification of mlrA-Genotype Microcystin-Degrading Bacteria

**Figure 2:**
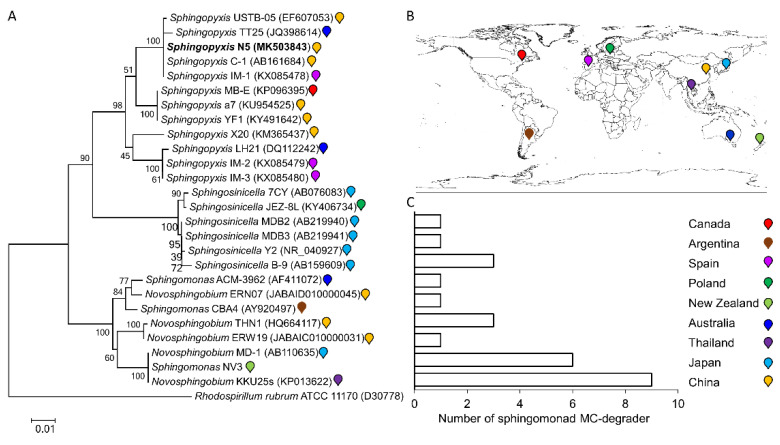
(**A**)16S rRNA-based phylogenetic analysis of the newly isolated indigenous MC degrader *Sphingopyxis* N5 (in bold) in northern Lake Taihu against 25 MC-degrading sphingomonad strains previously identified, *Rhodospirillum rubrum* ATCC 11170 was chosen as the outgroup. Local bootstrap probabilities were indicated at nodes. The access number for each strain’s 16S RNA was shown in brackets. The color-coding mark beside each bracket was used to indicate each strain’s origin. (**B**) The origin of the 26 microcystin-degrading sphingomonad strains. (**C**) The bar chart summarizing the number of the identified MC-degrading sphingomonad isolates in each country (China, Japan, Thailand, Spain, Poland, Canada, Argentina, Australia, New Zealand) across the Asia, Europe, North America, South America, and Oceania.

We further isolated multiple *mlrA-*genotype degraders from northern Lake Taihu. Five isolates (N5, THE1, TH2, TH3, and TH9) were picked at random, and grew round and smooth yellowish colonies on the agar plate, showing a rod shape with single polar flagella (Figure S2), in accordance with the morphological features of sphingomonads [29]. Their 16S rRNA sequences showed up to 99% nucleotide identity with that of *Sphingopyxis* sp. C-1, a *mlrA*-genotype degrader previously isolated from Lake Hongfeng, China [53], and also show high identity (98.3–98.5%) with that of a recently reported indigenous *Sphingopyxis* YF1 (*mlrA*-genotype) degrader isolated from Lake Taihu [12]. 16S rRNA-based phylogenetic analysis showed that new isolate N5 was located within a *Sphingopyxis* clade and clustered tightly with the *Sphingopyxis* degraders (C-1, TT25, IM-1, and USTB-05) (Figure 2A). Therefore, these five new isolates from Lake Taihu in this work were tentatively designated as *Sphingopyxis.*

The *mlrA* sequences within these five new degraders were almost identical (99–100% identity) and also highly similar to these of previously reported degraders (96–99% similarity), such as *Sphingopyxis* C-1, USTB-05, m6, a7, YMCD, MB-E, and YF1. Likewise, the MlrA amino acid sequences were highly similar among these strains (94–100% identity). Furthermore, the *mlr*-genotype *Sphingopyxis* degraders (such as *Sphingopyxis* C-1, a7, m6, USTB-05, YCDM, and YF1) have been frequently isolated from Lake Taihu and other Chinese sources [12,23,24,53,54] (Table 1). To date, *Novosphingobium* THN1, ERW19, and ERN07 that were isolated from northern Lake Taihu [21,55], so far, are the only three non-*Sphingopyxis* sphingomonad degraders reported in China (Figure 2A,B). Therefore, *Sphingopyxis* was likely to be the predominant *mlrA*-genotype degrader in Lake Taihu, and even in China, although other *mlrA*-genotype non-sphingomonad degraders (such as *Rhizobium* TH, *Bacillus* EMB, and *Stenotrophomonas* EMS) from the sediment, algal heap, and algal sludge of Lake Taihu were accidentally reported [22,56,57].

So far, there have been a total of 26 sphingomonad degraders that were molecularly identified and characterized. As shown in Figure 2C, the number of the isolates in Asia (*n* = 16) is much higher than those in other continents such as in Europe (*n* = 4), Oceania (*n* = 4), North America (*n* = 1), and South America (*n* = 1).

### 2.4. Degradative Capability and Specificity of Indigenous Bacterium

**Figure 3:**
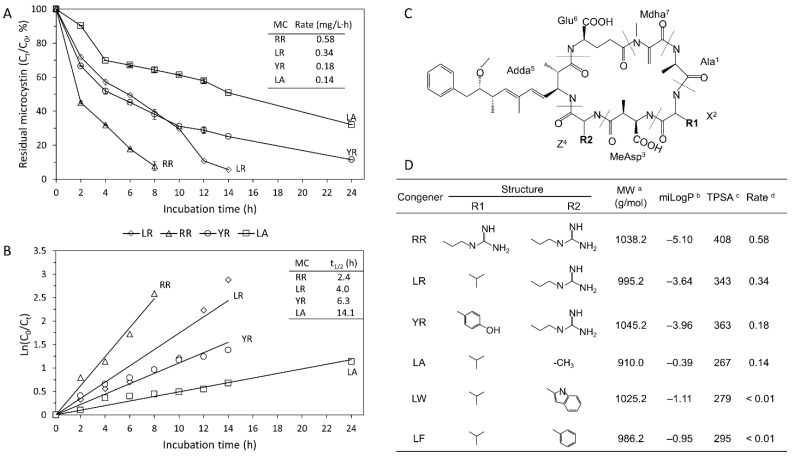
Characterization of microcystin-degrading capability and specificity of indigenous degrader *Sphingopyxis* sp. N5. (**A**) Degradation of four microcystin congeners (LR, RR, YR, and LA; 5 mg/L of each) by the degrader N5 in modified M9 broth at 28 °C, with microcystin as the sole carbon source. (**B**) Pseudo-first-order curve of microcystin degradation kinetics for LR, RR, YR, and LA. *C*_0_ denotes the initial concentration of microcystin; and *C_t_* and t_1/2_ denote the residual microcystin concentration at sampling time (t) and the degradation half-life of each variant, respectively. (**C**) The structure of micocystins. (**D**) The molecular weight (MW, g/mol), polarity-related parameters (topological polar surface area (TPSA), and miLogP) of six microcystin congeners (LR, RR, YR, LA, LF, and LW), and their degradation rates by degrader N5. ^a^ MW (molecular weight); ^b^ miLogP is a lipophilicity parameter; ^c^ TPSA is the topological polar surface area that is a polarity-related parameter; ^d^ Rate is the degradation rate of *Sphingopyxis* N5 for each microcystin congener.

The *Sphingopyxis* sp. strain N5 was selected at random. Only the six microcystin congeners (e.g., LR, RR, YR, LA, LF, and LW) were tested in this work, but not for the congener (e.g., [Dha^7^]LR) that was commercially unavailable) although it occurred in northern Lake Taihu [58]. As shown in Figure 3A, N5 was not only capable of degrading four microcystin congeners (LR, RR, YR, and LA), but also showed markedly differential degradation rates. Specifically, microcystin congeners (LR, RR, YR, and LA) at an initial concentration of 5 mg/L were removed 100%, 100%, 89%, and 68%, respectively, within 24 h at 28 °C (Figure 3A). The degradation rates of RR and LR (0.58 and 0.34 mg/(L·h), respectively) were higher than those of YR and LA (0.18 and 0.14 mg/(L·h), respectively). Modeling analysis showed that the kinetics of microcystin degradation fitted the first-order model well, with degradation half-lives of 2.4, 4.0, 6.3, and 14.1 h for RR, LR, YR, and LA, respectively (Figure 3B). Of note, no significant degradative activity within 24 h was observed for LF and LW that are rare in Lake Taihu.

Additionally, the substrate specificity pattern of strain N5 for microcystin congeners (RR > LR > YR > LA > LF, LW) was distinct from those of previously reported *mlrA*-genotype degraders such as *Sphingomonas* ACM3962 (LR > RR, degraded by cell extract) [5], *Novosphingobium* MD-1 (RR > YR > LR) [28], *Sphingosinicella* Y2 (YR > RR > LR) [27], *Sphingopyxis* MB-E (Approx. LR = RR = LF = LW) [33], and *Sphingosinicella* B-9 (LR = RR) [30].

To answer whether the microcystin degradation rate of *Sphingopyxis* N5 was associated with the weight or polarity (or lipophilicity) of the microcystin congener, we conducted correlation analysis; two polarity-(or lipophilicity)-related parameters (miLogP and topological polar surface area, TPSA) of microcystin variants were calculated using the online Molinspiration tool (https://www.molinspiration.com/ accessed on 1 February 2019). As a result, the microcystin degradation rate of *Sphingopyxis* N5 was not significantly correlated with the molecular weight or the polarity-related parameter (miLogP and TPSA) of microcystin congener (*p* > 0.05, Figure 3C,D).

### 2.5. Meta-Analysis of Microcystin Composition Profile in Northern Lake Taihu

**Figure 4:**
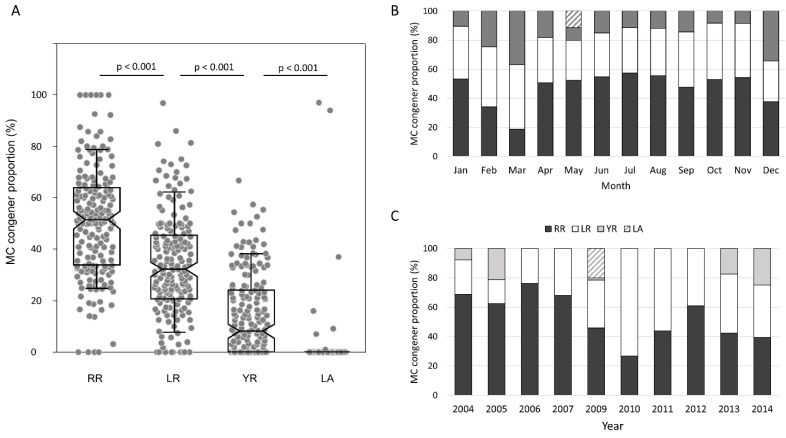
Meta-analysis of the composition pattern of microcystin congeners (LR, RR, YR, and LA) in northern Lake Taihu. (**A**) Box plot of the proportion values (%) of four microcystin variants (LR, RR, YR, and LA) in northern Lake Taihu according to the meta-analysis. (**B**) Temporal variation in the proportions of LR, RR, YR, and LA in northern Lake Taihu over 11 years (2004–2014), according to the meta-analysis. (**C**) Temporal variation in the proportions of LR, RR, YR, and LA in northern Lake Taihu over 12 months.

In this meta-analysis for the long-term profile of microcystin congener production in northern Lake Taihu, a total of 16 eligible publications were selected (listed in supporting material), involving 517 microcystin data points for algal samples from over 15 locations in the northern Lake Taihu from 2004 to 2014. The meta-analysis showed that RR was the most dominant congener, followed by LR, YR, and LA (Figure 4A). The average proportion of RR, LR, YR, and LA was 51%, 34%, 14%, and 1%, respectively, and the proportions of any two congeners were significantly different (*p* < 0.001, Mann–Whitney U test). Temporally, the microcystin composition profile was generally stable over 12 months or 11 years, still with RR the most dominant followed by LR (Figure 4B,C). The aforementioned substrate-specificity pattern was exactly congruent to the profile of microcystin congener production in northern Lake Taihu.

### 2.6. Meta-Analysis of Microcystin Degradation and Production Phenotype in the Natural Environment

To explore whether it was a global phenomenon that the substrate specificity pattern matched well the profile of microcystin congener production in a degrader habitat, we carried out a meta-analysis on global degraders. Notably, this work only focused on these degraders that have been shown to be able to degrade multiple microcystin congeners, and did not focus on the degraders (e.g., *Achromobacter* LG1 [59]) whose degradability of multiple MC congeners were not characterized yet. As a result, a total of 29 microcystin degraders were shown to degrade multiple microcystin congeners (Table 1). These comprised α-Proteobacteria (*Sphingopyxis*, *Sphingomonas*, *Sphingosinicella*, and *Novosphingobium*; *n* = 17), β-Proteobacteria (*Bordetella*, *Methylobacillus*, and *Paucibacter*; *n* = 3), γ-Proteobacteria (*Stenotrophomonas*; *n* = 2), Actinobacter (*Arthrobacter*, *Brevibacterium*, and *Rhodococcus*; *n* = 5), and Firmicutes (*Bacillus*; *n* = 2). These bacteria were isolated from China, Japan, Thailand, Spain, UK, New Zealand, Australia, Scotland, and Canada. Except for two water bodies (River Carron and Forfar Loch) [8], all other isolation sources experienced the occurrence of microcystin or toxic cyanobacterial blooms. 

Marked variability in the microcystin specificity pattern was observed among the global degraders (Table 1). For example, seven of the 29 degraders (24%) showed a higher degradation rate for RR than LR (RR > LR), while eight degraders (28%) showed a lower degradation rate for RR than LR (RR < LR) and four degraders (14%) showed similar degradation rates for LR and RR. Spatially, Asian sphingomonad degraders (such as *Sphingopyxis* N5 and *Sphingosinicella* B-9) typically showed greater degradation of the hydrophilic congeners (such as LR or RR) than the hydrophobic congeners (such as LF and LW) [30,44]. Conversely, Actinobacter degraders from Scotland (such as *Arthrobacter* C6 and *Rhodococcus* C1) showed higher degradation rates for the hydrophobic variants (such as LF and LW) than for hydrophilic RR and LR [60]. The Australian and Canadian sphingomonad degraders (e.g., *Sphingopyxis* TT25, and MB-E) showed similar degradation rates for hydrophobic or hydrophilic variants [33,34]. All these data together highlighted that the microcystin congener specificity pattern of the global degraders varied geographically. 

Further deep analysis against the profile of microcystin congener production in the degrader habitat showed that the degree of specificity for a certain microcystin congener exhibited by the degraders generally matched well with the degree of dominance of that congener in the degrader habitat. In other words, the microcystin degradation phenotype of the indigenous degrader was generally congruent to the microcystin production phenotype in the degrader habitat. Specifically, among the 13 cases for which both microcystin degradation and production profile data were available for analysis, eight cases (62%) showed congruence between microcystin degradation and production phenotype (Table 1). For example, *Sphingosinicella* 7CY showed a higher degradation rate for LR than for LF, LW, and LY (LR > LF, LW, and LY), which was exactly congruent to the profile of microcystin congener production in the 7CY habitat, Lake Suwa, Japan, where LR and RR were the major microcystin congeners [29,61]. Likewise, the degrader *Sphingosinicella* B-9 degraded LR, but not LF; the former was one of the major microcystin congeners in its habitat (Lake Tsukui, Japan) whereas the latter was not detected yet [62]. In cases where data for the microcystin composition pattern in the degrader habitat was lacking for comparison, we found that the microcystin degraders typically showed good degradation of common native microcystin congeners, rather than rare ones. For example, *Sphingopyxis* MB-E degraded microcystin congeners LF, LY, and LW as well as LR [33], all of which were common native microcystin congeners in the MB-E habitat Missisquoi Bay, Canada [63]. Similarly, Australian degrader *Sphingopyxis* LH21 degraded both LR and LA well, both of which were common in the Australian water body [35], the indigenous degrader *Sphingomonas* NV3 also degraded well LR and [Dha^7^]LR, which were the locally common congeners in the water bodies of New Zealand [36,64], the degrader *Noveosphingobium* MD-1 degraded well YR, RR, and YR, all of which were the common congeners in its habitat Lake Kasumigaura, Japan [28,65]. Two congeners LF and LW, which were rare in Lake Taihu, were not metabolized well by the indigenous degrader *Sphingopyxis* N5. Additionally, five incongruent cases were also observed in *Noveosphingobium* ERW19A and ERN07 [21], *Bordetella* MC-LTH1 from the algal sludge of Lake Taihu [66], *Sphingosinicella* Y2 from Lake Suwa, Japan [27], *Paucibacter* DSMZ-16998 from the Finnish Lake Tuusulanjarvi [60,67], *Paucibacter* DSMZ-16998 degraded the LF and LY much faster than RR that was one of the dominant congeners in the Lake Tuusulanjarvi [68]. Altogether these meta-analysis results highlighted that the microcystin degradation phenotype (or substrate-specificity pattern for microcystin congeners) of the microcystin degrader generally matched well the profile of microcystin congener production in the degrader habitat.

### 2.7. Analysis of Amino Acid Sequence of Partial Microcystinase MlrA against the Microcystin Congener Specificity

**Figure 5:**
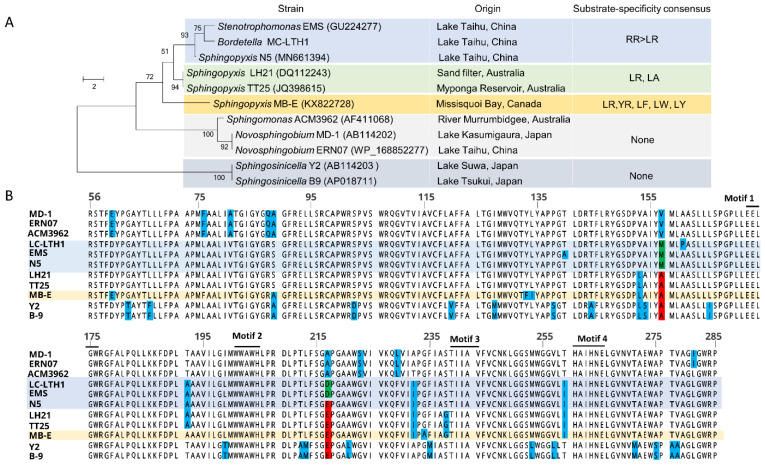
(**A**) (Left) Phylogenetic analysis of the translated amino acid sequences in the partial microcystinase MlrA (Position: 56–285) of 11 characterized microcystin degraders; the accession number of the corresponding *mlrA* sequences was shown in brackets, the *mlrA* sequence of *Bordetella* MC-LTH1 was retrieved from the reference [66]. (Middle) The middle column describes the origin of these 11 microcystin degraders. (Right) The right column described the consensus pattern of substrate specificity of the degraders. (**B**) Multiple-sequence alignment of the partial microcystinase MlrA homologs (Position: 56–285) in 11 microcystin degraders (the genus names were omitted). The amino acid residue at each position that shows dissimilarity with those in other strains was highlighted in various colors. Four motifs (Motif 1, EEXXXR; Motif 2, WXXXH, Motif 3, TXXXV; Motif 4, HXXHXE), which were previously assigned [71], were completely conserved through all the degraders and were marked under the line.

It is well known that the substrate specificity of the metabolizer was typically associated with the amino acid sequence, structure, and function of the degradation-related enzymes; for example, a minor change in the amino acids of the metabolic enzyme resulted in the significant alteration in substrate specificity of the metabolizer [47,48]. Therefore, we further investigated whether the substrate-specificity pattern of a certain microcystin degrader was associated with the amino acid sequence of the microcystinase MlrA, which is the first key degradative enzyme responsible for cleaving the cyclic microcystin into the linearized microcystin in the pathway of microcystin degradation [11,12,14,16], we selected a total of 11 *mlrA*-genotype microcystin degraders: *Sphingopyxis* N5, MB-E, LH21, TT25 (*n* = 7); *Spingosinicella* Y2 and B9 (*n* = 2); *Novosphingobium* MD-1 and ERN07 (*n* = 2), *Sphingomonas* ACM3962, *Stenotrophomonas* EMS, and *Bordetella* MC-LTH1 (Figure 5A), the sequences of their *mlrA* genes were partially or completely sequenced and the degradation rates for different microcystin congeners were also measured. Eventually, a 230-aa MlrA fragment (Position: 56–285) that was completely sequenced across all the 11 strains was focused upon. We observed (i) the amino acid sequences within these MlrA fragments were generally conserved with the identity of 87–100%, and the four putative motifs (Motif 1, EEXXXR; Motif 2, WXXXH, Motif 3, TXXXV; Motif 4, HXXHXE), which were previously assigned [71], were completely conserved through all the strains; (ii) of 230 positions, 37 residual positions (17%) were detected to harbor the alteration in amino acid sequence (Figure 5B); no single residual position was linked exclusively with the specific substrate-specificity pattern (RR > LR, or LR > RR, or RR = LR) except for the three residues (F^133^, I^134^, and A^234^) occurred uniquely in the Canadian degrader *Sphingopyxis* MB-E (Figure 5B), which also uniquely degraded these natively common congeners LR, YR, LA, LF, and LW at the same degradation rate [33]; (iii) more interestingly, three indigenous degraders of Lake Taihu (*Sphingopyxis* N5, *Bordetella* MC-LTH11, and *Stenotrophomonas* EMS), which consistently degraded RR faster than LR (Table 1), also had high similarity in MlrA sequences that were clustered within the same phylogenetic subclade (Figure 5A), moreover, two Australian strains LH21 and TT25 consistently degraded LR and LA well (Figure 5A), also showed the same 100% sequence amino identity. Taken together, these results highlighted the genotypic diversity of microcystinase MlrA sequences among global microcystin degraders, and also implied the association of the substrate specificity of the degrader with amino acid composition of MlrA. 

## 3. Discussion

Microbial degraders play an important role in reducing environmental microcystins. Here we addressed two important eco-physiological aspects of the *mlrA*-genotype degrader: (i) the ecological distribution pattern and (ii) the relationship between substrate specificity of indigenous microcystin degrader and the profile of microcystin congener production in the habitat. We showed that microcystin degraders (*mlrA*-genotype) were widely distributed in a large Chinese Lake Taihu and were closely associated with *Microcystis* abundance. Furthermore, through the individual analysis of an indigenous degrade in Lake Taihu and the meta-analysis of global microcystin degraders, we further identified the phenotypic congruence between the substrate specificity of microcystin degrader and profile of microcystin congener production in the degrader habitat, moreover, we also established the association of substrate specificity of the degrader with the sequence of microcystinase MlrA. We proposed the phenotypic congruence between microcystin degradation and production was associated with metabolic adaptation of degrader to the production of local microcystin congeners via genetic mutation in the degradative enzymes (e.g., microcystinase MlrA), and under the nutrient microcystin selection the indigenous microcystin degrader has evolved to better exploit these locally dominant microcystin congeners than the minor ones.

With the development of molecular technology, the ecological dynamics of *mlrA*-genotype degrader have been recently investigated in a few of locations of the lakes such as Lake Suwa, Japan [37,41], Lake Dianchi, China [42], and San Juan reservoir, Spain [43]. These field studies revealed the close association of microcystin degraders with microcystin concentration or *Microcystis* abundance. This work continued to emphasize that the co-occurrence of microcystin producer and degraders was a common ecological phenomenon worldwide. The underlying mechanisms involved at least three major factors: (i) as an important source of nutrient and energy, microcystins closely regulated the occurrence of microcystin degraders (*mlrA*-genotype). Previous studies have demonstrated that environmental microcystin concentrations significantly induced the increase in the abundance of microcystin degrader or *mlr* transcripts [23,42,55,72]; (ii) the mucilage of a *Microcystis* colony in a natural waterbody provides a beneficial habitat for microcystin degraders, Maruayama and colleagues observed that most microcystin degraders (*mlrA*-genotype) lived within the *Microcystis* colonies in the natural water body [37,41]; (iii) the exudates and extracellular macromolecules from *Microcystis* colony can constitute the additional diet sources that facilitate the growth of microcystin degraders [73]. Together, such unique diet and habitat-associated niches underlie the close co-occurrence of microcystin degraders with *Microcystis*.

By contrast with the widespread occurrence of sphingomonad microcysin degraders (*mlr*-genotype) in water bodies of China, Japan, Thailand, Australia, and New Zealand such as Lake Taihu, Lake Dianchi [42], Mongpa Reservoir [34], and Lake Suwa [27] as well as Bueng Nong Khot Reservoir [31], the isolation of *mlrA*-genotype degrader was occasionally reported in other continents such as Europe, North America, and South America. Dziga and colleagues showed the absence of *mlrA* gene in the studied samples from Poland [9,38]. The lack of *mlrA* gene was also consistently observed in the indigenous microcystin degrader in the Scottish water bodies [74], in the water body of central Poland [75], and in Lake Erie, North America [39,40]. Lezcano and colleagues showed that the *mlrA*-genotype degraders from the Spanish water samples accounted for minor fractions compared to the non-*mlrA* genotype ones [20]. Two possibilities might be responsible for such a discrepancy observed in these studies: (i) technically, the used molecular probes (e.g., MF/MR) were not degenerate oligonucleotides, not covering the variations of global *mlrA-*genotype degraders [21], and thus potentially generated the false negative detection; (ii) the dominance of *mlrA*-genotype degraders was dependent on geographical location. Recently, Wilhelm and colleagues analyzed the algal samples collected from Lake Taihu during the algal bloom period and the transcripts of only *mlrABC*-like genes, but not the *mlrABC*, were detected [76]. In future, more studies will yield a clear picture of *mlrABC* expression in the natural environments.

**Figure 6:**
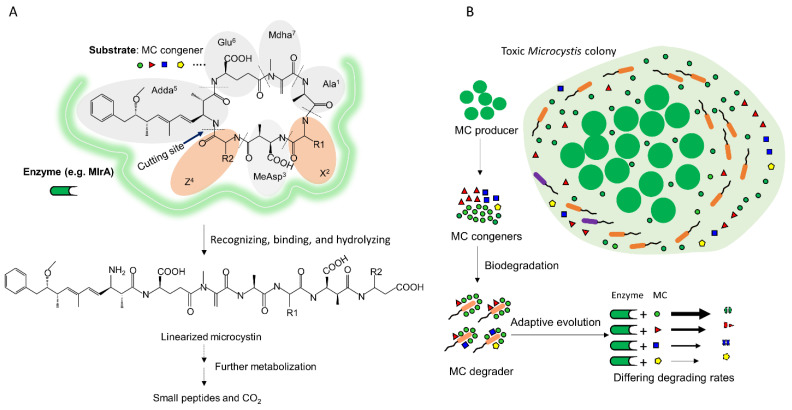
(**A**) The *mlr*-mediated pathway for degrading the MC congeners, which was illustrated based on the findings of previous studies [11,12,16]. (**B**) The conceptual model for the microbial adaption of MC degraders living within the mucilage of toxic *Microcystis* colony to the dominant MC congeners produced.

Toxic *Microcystis* simultaneously produces multiple microcystin congeners due to the relaxed specificity of the microcystin synthases [77]. Structurally, microcystin congeners share the core ring structure made of seven amino-acid residues (Ala, X, MeAsp, Z, Adda, Glu, and Mdha), X and Z represent two variable amino acids. MC-LR is the most common microcystin congener, in which X and Z are leucine (L) and arginine (R), respectively. Figure 6A illustrates the typical *mlr*-mediated pathway of microcystin degradation based on previous studies [11,12,13,14,16]. The structural variation of X and Z residues of microcystin congeners can affect the recognition or binding by the degradative enzyme, eventually can cause the disparity in the rates of degrading the congeners. The differential substrate-specificity patterns for microcystin congeners (e.g., LR, LF, or LA) have been previously confirmed in cell extract of *Sphingosinicella* B-9 [44], and in the *Sphingomonas* ACM-3962 [35]. This study further highlighted that the substrate-specificity patterns of global microcystin degraders were highly diverse and dependent on the geographical locations, and that the pattern of RR > LR was especially apparent for the indigenous degraders in Lake Taihu, China.

Two independent analyses (individual analysis of ingenious degrader in northern Lake Taihu and meta-analysis of global degraders) aided to establish that the substrate specificity of microcystin degrader was associated with the profile of microcystin congener production in the degrader habitat. The individual analysis on *Sphingopyxis* N5 revealed that the order of the degradation rates for microcystin congeners (RR > LR > YR > LA > LF, LW) statistically significantly corrected with neither the molecular weight nor polarity of the congeners, but was congruent to that of the dominance levels of these congeners (RR > LR > YR > LA > LF, LW) in northern Lake Taihu. To rule out the possibility of the coincidental congruence in a single strain, further meta-analysis of global degraders was conducted. The global microcystin degraders typically metabolized well these endemically dominant microcystin congeners, rather than the rare ones. This work had a limitation in the size of the indigenous strains characterized. In future, further investigation with more indigenous strains will provide the clearer picture.

Such a phenotypic congruent relationship was not a surprising ecological phenomenon. In the prior study, the substrate specificity has been theorized to result from the natural selection imposed by the availability of substrate [46]. This can be evidenced widely in the fields of microbial degradation of xenobiotics e.g., herbicides [78] and in the field of bacterial hydrolysis of antimicrobials (e.g., beta-lactam) in clinic [79]. As either the nutrient source or the toxic stressor, the substrates constitute a locally selective force that drives the bacteria to develop the specific enzymatic system for either metabolizing them or hydrolyzing them. Such scenario should hold true in the microbial degradation of microcystin congeners. Here we propose a conceptual model for microcystin metabolism of the microcystin degrader living within the *Microcystis* colony mucilage (illustrated in Figure 6A). We theorize that, as the important nutrient source in the *Microcystis* colony-mucilage microenvironment [37], the released microcystin congeners turn into the locally selective force for the bacteria living within the *Microcystis* colony. Over the process of adapting, the degradative pathway has been being evolved more specifically for the dominant microcystin congeners than the minor ones. As for the observed incongruence between microcystin degradation and production, these degraders showing incongruence might be not the major type of microcystin degraders. Therefore, the degradative phenotypes of indigenous microcystin degraders in a water body should be as diverse as the phenotype microcystin production in a water body [49,80].

Our present analysis revealed that the association of the substrate-specificity pattern of degrader with the sequences of microcystinase MlrA. How the microcystin-degrading enzymes govern the substrate specificity for microcystin congeners would be addressed in future.

This study has multiple implications: (i) the adaptive degradation observed in microcystin degrader might expand to the degraders of other algal toxins, the co-occurrence of algal toxin producer and degrader might be also universal; (ii) the differing substrate specificity for microcystin congeners implied that utilizing a mixture of microcystin degraders harboring different degradative phenotypes is more efficient than utilizing a single degrader to remove the complicated microcystin congeners; (iii) the differing substrate specificity can partially interpret the differing environment fates of microcystin congeners; (iv) the findings of this work also highlighted the ecological roles of microcystins in affecting the distribution and metabolism of bacteria, besides these widely discussed eco-physiological roles e.g., in maintaining *Microcystis* colonies [81], and modulating the Calvin cycle-associated protein RubisCo [82].

## 4. Conclusions

This study addressed two knowledge gaps regarding the ecological distribution and adaptive degradation of indigenous microcystin degraders. In Lake Taihu, microcystin-degrader (*mlrA*-genotype) was widespread in distribution and was closely associated with *Microcystis* abundance. Moreover, we established the existence of the phenotypic congruence between the substrate specificity of the degrader and the profile of microcystin congener production in the habitat. We theorized that such phenotypic congruence was associated with the metabolic adaptation of indigenous microcystin degraders to the complicated microcystin congeners in the habitat. These findings provide a novel insight into the ecophysiology of microcystin degrader and have important implications for understanding the biodegradation of algal toxins, evaluating the environmental fates of algal toxins, guiding efficient removal of algal toxins.

## 5. Materials and Methods

### 5.1. Field Survey Study Sites and Sampling

Lake Taihu is the third largest freshwater lake in China and located in the Jiangsu and Zhejiang provinces of southeast China. Lake Taihu is the key drinking water source for millions of local residents and is a typically shallow lake with an average depth and water area of 1.9 m and 2338 km^2^, respectively. In past decades, rapid development in both population and agro-industry in local catchment areas have caused substantial nutrient loadings in Lake Taihu, especially into the northern basin, and accelerated the eutrophication. In the summer of 2007, the outbreak of massive *Microcystis* blooms in Lake Taihu caused a significant water crisis in the local city of Wuxi that left millions of local residents without tap water for days [83].

A total of 110 samples were taken from 22 sites (N1–7, W1–6, and S1–9) across the entire Lake Taihu over 5 months from November 2008 to March 2009 (Figure 1A). Samples were collected as described previously [50]. Briefly, surface water samples (500 mL) were collected from depths of 0–0.5 m, and bacterioplankton samples (500 mL) were collected via horizontally towing the surface water using a phytoplankton net (No. 25; mesh size, 45 μm). A subsample (1000 mL) for each site was fixed with 1% Lugol’s iodine solution for phytoplankton identification and enumeration. Samples were stored in a cool container prior to analysis.

### 5.2. Measurement of Environmental Parameters

Routine physiochemical parameters (such as temperature, pH, and dissolved O_2_) were measured in situ using a water quality probe (YSI-550A Multiparameter Water Quality Sonde, YSI, Yellow Springs, OH, USA). The nutrients, chlorophyll *a*, and extracellular microcystin were determined and reported in our prior study [50]. Specifically, total phosphorus and nitrogen were measured according to two national standard methods by Chinese EPA: the ammonium molybdate spectrophotometric method (GB11893-89) and the alkaline potassium persulfate digestion-UV spectrophotometric method (GB11894-89), respectively. The chlorophyll *a* was measured using the acetone extraction- based spectrometric method [84]. The extracellular microcystins were determined using ELISA [85]. *Microcystis* was identified according the description by Komárek and Komárková (2002) [86], and enumerated using the counting chamber in a light microscope.

### 5.3. DNA Extraction and Detection of mlrA Genotype

The bacterioplankton samples were centrifuged, the upper and lower fractions (containing the floating algal cells and precipitation, respectively) were combined and suspended in lysis buffer (40 mM Tris-HCl, pH 8.0, 20 mM sodium acetate, 1 mM EDTA, 1% SDS). Genomic DNA was isolated using the phenol-chloroform protocol, suspended in Milli-Q water, and stored at −20 °C until further analysis. Detection of the *mlrA-*genotype microcystin degraders was performed by amplifying a genetic element of the *mlrA* gene (~0.8 kb) using *mlrA*-specific primer set MF/MR [18]. The PCR products were applied to agarose gel electrophoresis and visualized on a UV transilluminator.

### 5.4. Isolation and Identification of Indigenous Microcystin Degraders (mlrA-Genotype)

Isolation of microcystin degraders was carried out between 2008 and 2009 according to previously reported methods [29,35], with a slight modification. Briefly, a water sample with a positive PCR-*mlrA* screening result was inoculated into the selective mineral medium supplemented with microcystin extract from *Microcystis*. After incubation for 3 days at 28 °C, the culture was spread onto a Luria broth (LB) agar plate. The *mlrA*-positive colonies that are potential microcystin degraders were selected and purified. Molecular identification of the *mlrA*-genotype degrader was performed by amplifying and sequencing 16S rRNA using universal primer set 27F/1492R [87], and the partial *mlrA* fragment from the PCR using the primer set MF/MR [18].

### 5.5. Characterization of Microcystin Degradation by Indigenous Degrader

An indigenous degrader (*Sphingopyxis* N5) isolated from the sampling site N3 in northern Lake Taihu was chosen at random to characterize the biodegradability of indigenous degraders in northern Lake Taihu. A total of six different MC congeners (MC-LR, MC-RR, MC-YR, MC-LA, MC-LW, and MC-LF, further short for LR, RR, YR, LA, LW, and LF in this work, respectively; Abraxis Company, Los Angeles, CA, USA) were chosen for the degradation assay. Briefly, *Sphingopyxis* N5 culture at the exponential phase was harvested and washed with glucose-free M9 broth three times to remove the residual carbon source [29]. The washed culture was inoculated into the modified M9 broth that contained only a single type of MC congener (5 mg/L) as the sole carbon source in each individual MC congener assay, the initial concentration of inoculum in the broth and the incubation temperature were 1 × 10^6^ cells/mL and 28 °C, respectively. Samples were taken at 0, 2, 4, 6, 8, 10, 12, 14, and 24 h. Residual microcystin congener in the broth of each individual assay was quantified by high-performance liquid chromatography (HPLC) on a Shimadzu LC-10A system equipped with a UV detector and Shimadzu shim-pack column (CLO-ODS 6.0 mm × 150 mm). The mobile phase was methanol/0.05 M K_2_HPO_4_ buffer (57:43, *v*/*v*; pH 3.0) at a flow rate of 1 mL/min, and the detection wavelength was set at 238 nm [88], the limit of detection in the HPLC instrument was 0.1 μg/mL, the recovery and the relative precision of the method were > 80% and < 5%, respectively. The experiment was conducted in duplicate. The kinetics of microcystin degradation by *Sphingopyxis* N5 were simulated using a pseudo-first-order degradation model. Equations (1) and (2) were used to evaluate the degradation rate constant (k, h^−1^) and half-life (t_1/2_), respectively:

(1) $$\ln\frac{C_{t}}{C_{0}}=-\mathrm{kt}$$

(2) $$t_{1/2}=\frac{\ln2}{k}$$

where C_t_ and C_0_ are the residual microcystin concentrations at sampling time t (h) and the starting time (0 h), respectively, and k is the rate constant of microcystin removal (h^−1^).

### 5.6. Meta-Analysis of Perennial Microcystin Composition in Northern Lake Taihu

Although the production of microcystin congeners in northern Lake Taihu has been extensively documented [50,89], the perennial microcystin composition pattern on a large spatial scale over the decade is lacking but is necessary for studying the long-term adaptation of indigenous degraders to the local microcystin production. To address this gap, we carried out a meta-analysis on the previously reported data of microcystin production in northern Lake Taihu. The entire meta-analysis procedure is shown in Figure S3. Briefly, a literature search of the ISI Web of Knowledge and PubMed databases was performed to identify relevant studies involving the quantification of microcystin variants in algal samples from northern Lake Taihu. No restriction was imposed on the time of publication. Studies were considered eligible if they provided both the sampling time and microcystin congener quantification data. The data were then extracted from selected studies, including quantification data for microcystin congeners, sampling location, and sampling time. If microcystin data were only graphically presented, the figures were imported into Microsoft PowerPoint and numerical values were manually estimated by comparing the relative distance of each datum point along the *x* or *y* axis with the corresponding scale on the axis. After data extraction, the profile of microcystin composition in each sample was evaluated by quantifying the proportion of each microcystin congener in total microcystins.

### 5.7. The Meta-Analysis of Microcystin Substrate Specificity of Global Microcystin Degraders

A meta-analysis was conducted to elucidate the relationship between the substrate specificity of the previously reported microcystin degraders worldwide and the profiles of microcystin congener production in the corresponding degrader habitats. A literature search for previously reported microcystin degraders was performed using the ISI Web of Knowledge and PubMed databases as well as reference lists (if applicable). The data retrieved comprised the degrader taxon name, isolation origin (habitat), and the degradability of the degrader microcystin for various microcystin congeners (LR, RR etc.), as well as the microcystin congener production (composition) profile in the degrader habitat, the latter being obtained from a further literature search. The pattern of substrate specificity of microcystin degrader was evaluated via comparing the microcystin degradation-associated parameters e.g., average degradation rate (mg/L·h), degradation constant (h^−1^), half-life (t_1/2_, h or d) directly shown or indicated in the literature. The profile of microcystin congener production in the degrader habitat was characterized by comparing the relative dominance levels of microcystin congeners shown in the literature. To avoid severe bias, the difference in the degradation rate of each studied congener was not compared if the degradation assays of a certain degrader for different microcystin congeners were performed in different experimental conditions, or if the difference in the initial concentrations of different congeners in the degradation assay exceeded 10 folds.

### 5.8. Construction of Phylogenetic Tree and Homology Analysis of MlrA Amino Acid Sequences

The 16S rRNA nucleotide sequences of five microcystin degraders (N5, THE1, TH2, TH3, and TH9) were queried in the NCBI database using BLASTn. Phylogenetic trees were built using the neighbor-joining method based on 1000 bootstrap replicates with MEGA7 software (Center for Evolutionary Medicine and Informatics, Tempe, AZ, USA). Homology analysis of partial MlrA amino acids sequences of microcystin degraders were conducted using the Clustwal Omega. The MlrA-associated phylogenetic tree was built using the neighbor-joining method with MEGA7 software. The 16S rRNA and *mlrA* sequences of five degraders isolated in this work were deposited in GenBank under accession numbers MK503843 to MK503847 (16S rRNA), and MN661394 to MN661398 (*mlrA*).

### 5.9. Statistical Analysis

Differences in the routine physiochemical parameters (temperature, pH, and dissolved oxygen), extracellular microcystin concentration, and *Microcystis* abundance between PCR-*mlrA*-positive and PCR-*mlrA*-negative samples were evaluated using a two-sample *t*-test. Differences in the proportion of microcystin variants in the meta-analysis were evaluated using the Mann–Whitney U test. Correlation between microcystin degradation rate and the chemical parameters (molecular weight, TPSA, and miLogP) of microcystin was evaluated using Pearson correlation analysis. All statistical analyses were conducted using the NCSS 11.0 software (NCSS, LLC, Kaysville, UT, USA) with the significance level set at *p* < 0.05.

## Figures and Tables

**Table 1:** Analysis of the relationship between substrate specificity patterns of the microcystin (MC) degraders (*n* = 29) and the MC production profiles in the degrader habitats.

Strain	Location	MC Production in Degrader Habitat		MC Degradation of Indigenous Degrader				^b^ Congruence
		Profile	Reference	Substrate Specificity	^a^ Degradation Rate (mg/(L·h))	Initial MC Concentration (mg/L)	Reference	
*Sphingopyxis* N5	Northern Lake Taihu, China	RR > LR > YR > LA	This study	RR > LR > YR > LA > LF, LW	RR (0.58), LR (0.34), YR (0.18), LA (0.14)	Each MC (5)	This study	Yes
*Methylobacillus* J10	Algal sludge from Northern Lake Taihu, China	RR > LR > YR > LA	This study	RR > LR	RR (>0.24), LR (<0.24)	RR (~4.3), LR (~3.5)	[69]	Yes
*Bordetella* MC-LTH1	Algal sludge from Lake Taihu, China	RR > LR > YR > LA	This study	RR < LR	LR (0.31), RR (0.17)	RR (5), LR (11)	[66]	No
*Stenotrophomonas* EMS	Algal sludge from Northern Lake Taihu, China	RR > LR > YR > LA	This study	RR > LR	RR (0.07), LR (0.03)	RR (1.7), LR (0.7)	[22]	Yes
*Stenotrophomonas* MC-LTH2	Algal sludge from Lake Taihu, China	RR > LR > YR > LA	This study	RR > LR	RR (0.23), LR (0.125)	LR (21.2), LR (39.2)	[26]	Yes
*Bacillus* EMB	Algal heap from Northern Lake Taihu, China	RR > LR > YR > LA	This study	RR > LR	RR (0.125), LR (0.09)	RR (2.99), LR (2.15)	[56]	Yes
*Novosphingobium* ERW19	Northern Lake Taihu, China	RR > LR > YR > LA	This study	RR = LR	RR (0.008), LR (0.008)	Each (0.1)	[21]	No
*Novosphingobium* ERN07	Northern Lake Taihu, China	RR > LR > YR > LA	This study	RR = LR	RR (0.005), LR (0.005)	Each (0.1)	[21]	No
*Sphingopyxis* USTB-05	Lake Dianchi, China	RR > LR	[50]	LR, RR, YR	LR (1.2), RR (0.7), YR (1.48, by enzyme) in different conditions	RR (50.2), LR (28.8),YR (14.8)	[14,24,25]	-
*Sphingopyxis* C1	Lake Hongfeng, China	RR > LR	[70]	LR, RR	LR (0.33), RR (^e^ n.a)	LR (1), RR (^e^ n.a.)	[53]	-
*Sphingosinicella* Y2	Lake Suwa, Japan	LR, RR > YR	[61]	YR > RR > LR	YR (2.5), RR (0.54), LR (0.23)	YR (22), RR (18), LR (18)	[27]	No
*Sphingosinicella* 7CY	Lake Suwa, Japan	LR, RR > YR	[61]	LR > LF = LW = LY	LR (t_1/2_, ~2.4d), LF, LW, LY (t_1/2_, ~3.3d)	Each (6)	[29]	Yes
*Sphingosinicella* B-9	Lake Tsukui, Japan	LR, RR, YR	[62]	LR = RR > LF	LR, RR (0.08), LF (undegraded by cell extract)	LR (2), RR (2), LF (91)	[30,44]	Yes
*Novosphingobium* MD-1	Lake Kasumigaura, Japan	LR, YR, RR	[65]	YR > RR > LR	YR (t_1/2,_ 1.9 h^−1^), RR (1.25 h^−1^), LR (0.66 h^−1^)	Each (1)	[28]	-
*Rhodococcus* C1	River Carron, Scotland	no MCs	[8]	LY > LW > LF > LR > RR	LY (t_1/2_, 5d), LW (t_1/2_, 6d), LF (t_1/2_, 8d), LR (t_1/2_, 9d), RR (t_1/2_, 10d)	Each (10)	[60]	-
*Arthrobacter* C6	River Carron, Scotland	no MCs	[8]	LY > LF = LW > LR > RR	LY (t_1/2_, 5d), LW (t_1/2_, 8d), LF (t_1/2_, 8d), LR (t_1/2_, 9d), RR (t_1/2_, >10d)	Each (10)	[60]	-
*Brevibacterium* F3	Forfar Loch, Scotland	no MCs	[8]	LY > LF > LR = LW > RR	LY (t_1/2_, 6d), LF (t_1/2_, 7d), LW (t_1/2_, 9d), LR (t_1/2_, 9d), RR (t_1/2_, >10d)	Each (10)	[60]	-
*Arthrobacter* F7	Forfar Loch, Scotland	no MCs	[8]	LY = LF > LW > LR > RR	LY (t_1/2_, 6d), LF (t_1/2_, 6d), LW (t_1/2_, 7d), LR (t_1/2_, 9d), RR (t_1/2_,>10d)	Each (10)	[60]	-
*Arthrobacter* R4	Loch Rescobie, UK	MCs (unknown)	[8]	LW > LY = LF > LR > RR	LW (t_1/2_, 6d), LY (t_1/2_, 7d), LF (t_1/2_, 7d), LR (t_1/2_, 9d), RR (t_1/2_, >10d)	Each (10)	[60]	-
*Paucibacter* DSMZ-16998	Lake Tuusulanjarvi, Finland	^c^ DmRR, RR etc.	[68]	LY = LF > LW > LR > RR	LY (t_1/2_, 6d), LF (t_1/2_, 6d), LW (t_1/2_, 7d), LR (t_1/2_, 9d), RR (t_1/2_, >10d)	Each (10)	[60]	No
*Sphingopyxis* IM-1	San Juan Dam, Spain	LR > RR > YR	[20]	^e^ RR, LR	RR (t_1/2_, < 2 h), LR (t_1/2_, > 2 h)	LR (0.92), RR (0.04), YR (0.04)	[20]	-
Sphingopyxis IM-2	San Juan Dam, Spain	LR > RR> YR	[20]	^e^ RR, LR	LR, RR (t_1/2_, ~110 h)	LR (0.92), RR (0.04), YR (0.04)	[20]	-
*Sphingopyxis* IM-3	San Juan Dam, Spain	LR> RR> YR	[20]	^e^ RR, LR	RR (t_1/2_, ~20 h), LR (t_1/2_, ~30 h)	LR (0.92), RR (0.04), YR (0.04)	[20]	-
*Paucibacter* IM-4	San Juan Dam, Spain	LR> RR> YR	[20]	^e^ RR, LR	LR, RR (t_1/2_, ~35 h)	LR (0.92), RR (0.04), YR (0.04)	[20]	-
*Sphingopyxis* MB-E	Missisquoi Bay, Canada	LR, YR, LY, LW, LF	[63]	LR = YR = LY = LW = LF	LR, YR, LY, LW, LF (6.25 × 10^−4^)	Each (0.01)	[33]	-
*Sphingopyxis* LH21	Sand filter, Australia	^d^ n.a.	-	LR = LA	LR, LA (1 × 10^−4^)	Each (0.01)	[35]	-
*Sphingomonas* ACM-3962	River Murrumbidgee, Australia	LR (major)	[5]	LR > RR, LA	LR (1.6) and RR (1.5) by cell free extract	Each (10)	[5]	Yes
					LA (5 × 10^−5^) by bacteria in reservoir water	LA (0.01)	[35]	
*Sphingopyxis* TT25	Reservoir Myponga, Australia	^e^ n.a.	-	LR = RR = YR = LA	LR, RR, YR, LA (2 × 10^−4^)	Each (0.01)	[34]	-
*Sphingomonas* NV3	Lake Rotoiti, New Zealand	LR, RR. etc.	[64]	LR, [Dha^7^] LR	Mixture of LR and Dha^7^ LR (0.35)	Mixture (25)	[36]	-

^a^ Instead of the degradation rate (mg/(L·h)), the half-life (t_1/2_, d) was provided to compare the degradation rates of microcystin congeners for the strains (C1, C6, F3, F7, R4, dsmz-16998, IM-1, IM-2, IM-3, IM-4, and 7CY), and the specific degradation constant (h^−1^) was provided for strain *Novosphingobium* MD-1. ^b^ Congruence is qualified by comparing the matching level between substrate specificity and the microcystin production profile in the degrader habitat. ^c^ DmRR, demethylated MC-RR, ^d^ n.a. not available. ^e^ The substrate-specificity patterns of the Spanish degraders (IM-1, IM-2, IM-3) for RR and LR were not compared because of the remarked difference (>20 folds) in the initial concentration of substrate (LR and RR) in the degradation assays.

## Data Availability

The 16S rRNA and *mlrA* sequences of five degraders isolated in this work were deposited in GenBank under accession numbers MK503843 to MK503847 (16S rRNA), and MN661394 to MN661398 (*mlrA*).

## Supplementary Materials

The following are available online at https://www.mdpi.com/article/10.3390/toxins13120864/s1: The list for the references used in the meta-analysis of the profile of MC congener production in northern Lake Taihu. Figure S1: (A) Frequency of PCR-mlrA-positive detection in each site of the northern and southern Lake Taihu. (B) The average frequency of PCR-mlrA-positive detection in the northern and southern lake sites. Figure S2: TEM photograph of the isolate Sphingopyxis N5. Figure S3: The workflow of meta-analysis on the profile of MC congener production in the northern Lake Taihu.
