# Supplementary material for: Widespread Distribution and Adaptive Degradation of Microcystin Degrader (mlr-Genotype) in Lake Taihu, China
Source: Toxins (Basel). 2021 Dec 3;13(12):864. doi: 10.3390/toxins13120864 (PMC8705652; doi:10.3390/toxins13120864)
Supplement: Supplementary file 1 [file toxins-13-00864-s001.zip › toxins-1470142-supplementary.pdf]

toxins

MDPI

Supplementary Materials: Widespread distribution and adaptive degradation of microcystin decomposer (*mlr*-genotype) in Lake Taihu, China

Chenlin Hu, Yanxia Zuo, Liang Peng, Nanqin Gan and Lirong Song

The list for the references used in the meta-analysis of the profile of MC congener production in northern Lake Taihu.

The meta-analysis of the profile of MC congener production in the northern Lake Taihu was based on previous studies (Chen *et al.*, 2006, Chen & Xie, 2007, Song *et al.*, 2007, Wu *et al.*, 2009, Zhang *et al.*, 2009, Liu *et al.*, 2011, Wilhelm *et al.*, 2011, Chen *et al.*, 2012, Ye *et al.*, 2014, Hu *et al.*, 2016, Jia *et al.*, 2016, Wei *et al.*, 2016, Xue *et al.*, 2016, Xue *et al.*, 2016, Su *et al.*, 2017, Su *et al.*, 2018).

Chen J & Xie P (2007) Microcystin accumulation in freshwater bivalves from Lake Taihu, China, and the potential risk to human consumption. *Environ Toxicol Chem* **26**: 1066-1073.

Chen J, Xie P, Zhang DW, Ke ZX & Yang H (2006) In situ studies on the bioaccumulation of microcystins in the phytoplanktivorous silver carp (Hypophthalmichthys molitrix) stocked in Lake Taihu with dense toxic Microcystis blooms. *Aquaculture* **261**: 1026-1038.

Chen W, Jia YL, Li EH, Zhao S, Zhou QC, Liu LM & Song LR (2012) Soil-Based Treatments of Mechanically Collected Cyanobacterial Blooms from Lake Taihu: Efficiencies and Potential Risks. *Environ Sci Technol* **46**: 13370-13376.

Hu LL, Shan K, Lin LZ, Shen W, Huang LC, Gan NQ & Song LR (2016) Multi-Year Assessment of Toxic Genotypes and Microcystin Concentration in Northern Lake Taihu, China. *Toxins* **8**.

Jia JM, Chen QW & Lauridsen TL (2016) A Systematic Investigation into the Environmental Fate of Microcystins and The Potential Risk: Study in Lake Taihu. *Toxins* **8**.

Liu YM, Chen W, Li DH, Huang ZB, Shen YW & Liu YD (2011) Cyanobacteria-/cyanotoxin-contaminations and eutrophication status before Wuxi Drinking Water Crisis in Lake Taihu, China. *J Environ Sci* **23**: 575-581.

Song LR, Chen W, Peng L, Wan N, Gan NQ & Zhang XM (2007) Distribution and bioaccumulation of microcystins in water columns: A systematic investigation into the environmental fate and the risks associated with microcystins in Meiliang Bay, Lake Taihu. *Water Res* **41**: 2853-2864.

Su XM, Steinman AD, Xue QJ, Zhao YY & Xie LQ (2018) Evaluating the contamination of microcystins in Lake Taihu, China: The. application of equivalent total MC-LR concentration. *Ecol Indic* **89**: 445-454.

Su XM, Steinman AD, Tang XM, Xue QJ, Zhao YY & Xie LQ (2017) Response of bacterial communities to cyanobacterial harmful algal blooms in Lake Taihu, China. *Harmful Algae* **68**: 168-177.

Wei N, Hu LL, Song LR & Gan NQ (2016) Microcystin-Bound Protein Patterns in Different Cultures of Microcystis aeruginosa and Field Samples. *Toxins* **8**.

Wilhelm SW, Farnsley SE, LeCleir GR, Layton AC, Satchwell MF, DeBruyn JM, Boyer GL, Zhu GW & Paerl HW (2011) The relationships between nutrients, cyanobacterial toxins and the microbial community in Taihu (Lake Tai), China. *Harmful Algae* **10**: 207-215.

Wu XQ, Xiao BD, Li RH, Wang Z, Chen XG & Chen XD (2009) Rapid quantification of total microcystins in cyanobacterial samples by periodate-permanganate oxidation and reversed-phase liquid chromatography. *Anal Chim Acta* **651**: 241-247.
Xue QJ, Steinman AD, Su XM, Zhao YY & Xie LQ (2016) Temporal dynamics of microcystins in Limnodrilus hoffmeisteri, a dominant oligochaete of hypereutrophic Lake Taihu, China. *Environ Pollut* **213**: 585-593.
Xue QJ, Su XM, Steinman AD, Cai YJ, Zhao YY & Xie LQ (2016) Accumulation of microcystins in a dominant Chironomid Larvae (Tanypus chinensis) of a large, shallow and eutrophic Chinese lake, Lake Taihu. *Sci Rep-Uk* **6**.
Ye R, Shan K, Gao HL, Zhang RB, Xiong W, Wang YL & Qian X (2014) Spatio-Temporal Distribution Patterns in Environmental Factors, Chlorophyll-a and Microcystins in a Large Shallow Lake, Lake Taihu, China. *Int J Env Res Pub He* **11**: 5155-5169.
Zhang D, Xie P, Liu Y & Qiu T (2009) Transfer, distribution and bioaccumulation of microcystins in the aquatic food web in Lake Taihu, China, with potential risks to human health. *Sci Total Environ* **407**: 2191-2199.

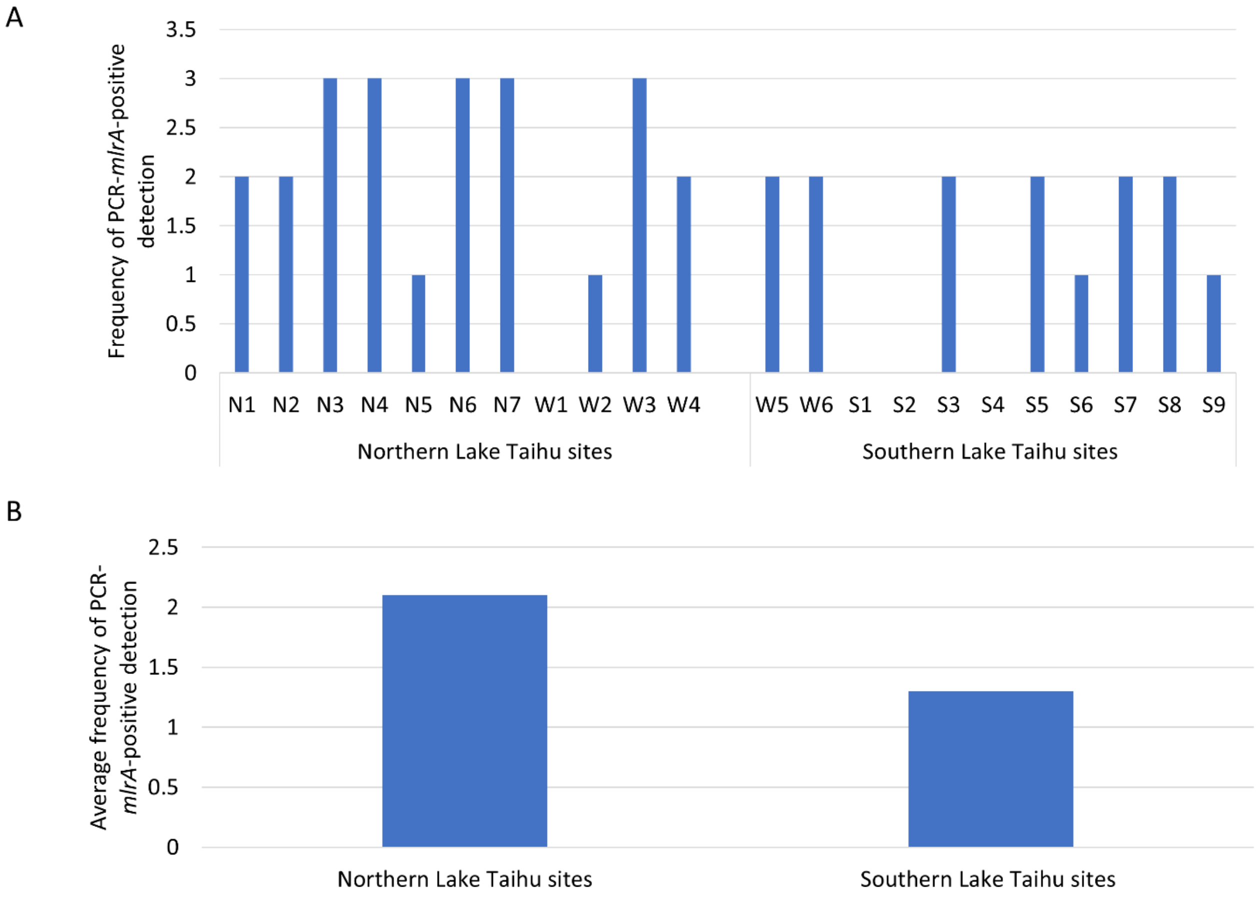

Figure S1. (A) Frequency of PCR-*mlrA*-positive detection in each site of the northern and southern Lake Taihu. (B) The average frequency of PCR-*mlrA*-positive detection in the northern and southern lake sites.

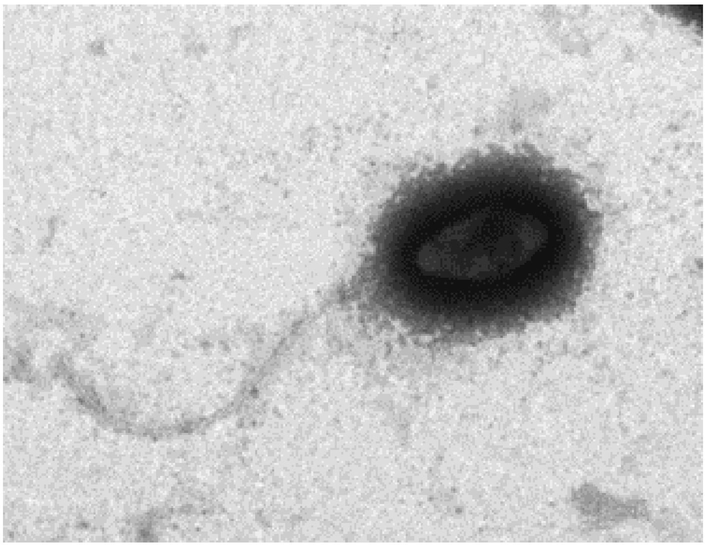

Figure S2. TEM photograph of the isolate *Sphingopyxis* N5.

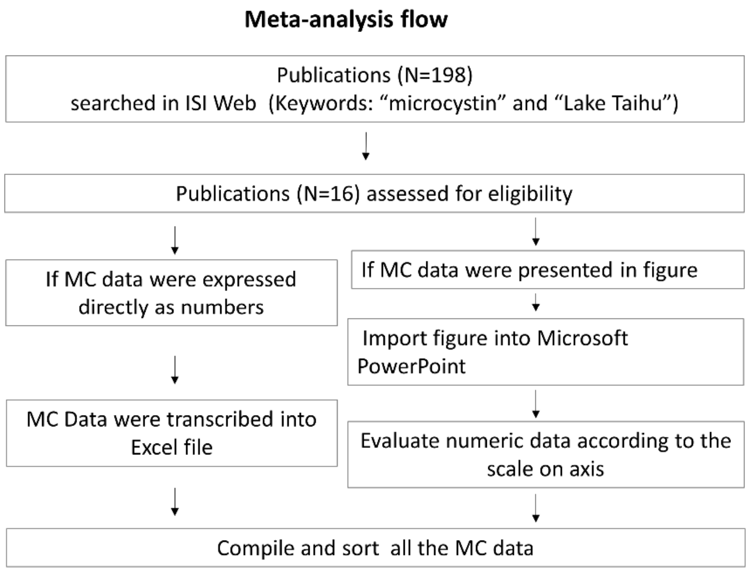

Figure S3. The workflow of meta-analysis on the profile of MC congener production in the northern Lake Taihu.
